# Editorial: Smart urban environmental health from multi-scale, multimedia, multi-exposure, multi-target perspectives

**DOI:** 10.3389/fpubh.2022.989922

**Published:** 2022-09-27

**Authors:** Fei Li, Chuanrong Zhang, Min Chen, Hongtao Yi

**Affiliations:** ^1^Research Center for Environment and Health, Zhongnan University of Economics and Law, Wuhan, China; ^2^Department of Geography, Center for Environmental Sciences and Engineering, University of Connecticut, Stamford, CT, United States; ^3^Key Laboratory of Virtual Geographic Environment (Ministry of Education of PRC), Nanjing Normal University, Nanjing, China; ^4^John Glenn College of Public Affairs, The Ohio State University, Columbus, OH, United States

**Keywords:** multimedia environmental risk, environmental monitoring, health geography, urban modeling and simulation, exposure management

Over half of the global population live in urbanized areas. These areas have become the geographic focus of resource consumption and chemical emissions. Pollutants in the urban environmental multi-media (including water, soil, air, etc.) are intensifying, causing chronic public health risk and hazard increases *via* multi-exposure pathways and multi-scale distribution differences. This Research Topic aims to provide a platform for researchers committed to the related progress on urban environmental health and sustainable development. As a result, our Research Topic has generated a great deal of interests and generated 11 multi-disciplinary articles in total.

The environmental monitoring and risk management research covered within this Research Topic includes studies about atmospheric pollution, soil pollution and cosmetic toxin. Contributors included a team from Chinese Academy for Environmental Planning published their systematic risk assessment in a typical contaminated site of a hazardous waste disposal center based on soil boreholes and groundwater monitoring wells investigation (Zhu et al.). Based on the chemical element analysis of lead (Pb) in the selected 34 popular lip cosmetics from Chinese e-commerce market, Li et al. found that there was no significant (non-)carcinogenic risk and blood Pb risk caused by adults and children's exposure to those lip cosmetics. The study by Zhu et al. proposed a hierarchical urban PM2.5 management policy by exploring the PM2.5 spatiotemporal evolution and its socioeconomic driver analysis. Further, the provincial baseline of PM2.5 in China was calculated by Jin et al., and their findings may help decision-makers to establish the differentiated control rules based on the classified cities.

The urban environmental health and sustainable development covered in this Research Topic also comprises a series of interdisciplinary studies, which explore this topic using methods from econometrics, landscape ecology, and environmental hygiene. The study by Chen et al. found that the economic losses of high temperature on the health of Wuhan residents were 156.1 billion RMB (95% CI: 92.28–211.40 billion RMB) during 2013–2019, accounting for 1.81% (95% CI: 1.14–2.45%) of Wuhan's annual GDP. Further, the findings of Liu et al. may help planners and government to understand the current cooling condition of green spaces, improve their cooling capacity, mitigate the urban heat island effect, and create a comfortable and healthy thermal environment during summer. Shan et al. analyzed the coordinated relationship between urban population–land spatial patterns (UPLSPs) and ecological efficiency (EE) in 12 Hubei cities as case studies. Their results indicated that the related departments should coordinate human and land resources and the ecological environment, and narrow regional development differences. By using the difference-in-differences (DID) method, Ye et al. found that the Ecological Red Lines (ERL)'s pilot scheme in four provinces of China hardly drove any promotion effects on the ratio of the tertiary industry to secondary industry while the residents' health was significantly improved by 1.029%. The study by Liu et al. analyzed the spatio-temporal variation of health production efficiency (HPE) of each province across China and highlighted the significant socioeconomic driving factors on health production efficiency, then targeted policies to accelerate the overall HPE development were proposed.

In this Research Topic, the environmental health law and geography studies have also been covered. The research by Zhang et al. highlighted the importance of knowledge on analyzing the subject and the subjective, incidence, and sentencing factors of wildlife crimes from legal and ethical perspectives, and uses the ecological economic ethical model to measure wildlife crimes. Cao et al. proposed a combining long short-term memory (LSTM) and quantum particle swarm optimization (QPSO) method to forecast the demand for shared bicycles in different urban regions, through which to reduce chemical emissions and improve public health by increasing user experiences.

The high diversity of the urban environment and public health studies on sustainability theme is likely to be of interest to a broad audience for upcoming decades. This Research Topic provides not just a timely reference source for academics, but also practical use for decision-makers, environmental engineers, and land planners concerned with environment-public health-sustainable development.

It is a pleasure to thank the members of the Editorial Board, all authors and co-authors and all referees for their valuable contributions to this Research Topic. Furthermore, these interesting publications would not be possible without the efficient support from the Journal Office.

## Author contributions

FL wrote the first draft of this manuscript. CZ, MC, and HY critically reviewed and approved the final paper. All authors contributed to the article and approved the submitted version.

## Conflict of interest

The authors declare that the research was conducted in the absence of any commercial or financial relationships that could be construed as a potential conflict of interest.

## Publisher's note

All claims expressed in this article are solely those of the authors and do not necessarily represent those of their affiliated organizations, or those of the publisher, the editors and the reviewers. Any product that may be evaluated in this article, or claim that may be made by its manufacturer, is not guaranteed or endorsed by the publisher.

